# How does comorbidity affect cost of health care in patients with irritable bowel syndrome? A cohort study in general practice

**DOI:** 10.1186/1471-230X-10-31

**Published:** 2010-03-17

**Authors:** Per A Johansson, Per G Farup, Andrea Bracco, Per O Vandvik

**Affiliations:** 1 Department of Medicine, Innlandet Hospital Trust, Gjøvik, Norway; 2 Unit for Applied Clinical Research, Norwegian University of Science and Technology, Trondheim; Norway; 3 Institute for Medical Technology Assessment, Erasmus University, The Netherlands

## Abstract

**Background:**

Irritable bowel syndrome (IBS) is associated with other disorders (comorbidity), reduced quality of life and increased use of health resources. We aimed to explore the impact of comorbidity on cost of health care in patients with IBS in general practice.

**Methods:**

In this cohort study 208 consecutive patients with IBS (Rome II) were recruited. Sociodemographic data, IBS symptoms, and comorbidity (somatic symptoms, organic diseases and psychiatric disorders) were assessed at baseline. Based on a follow up interview after 6-9 months and use of medical records, IBS and non-IBS related health resource use were measured as consultations, hospitalisations, use of medications and alternative health care products and sick leave days. Costs were calculated by national tariffs and reported in Norwegian Kroner (NOK, 1 EURO equals 8 NOK). Multivariate analyses were performed to identify predictors of costs.

**Results:**

A total of 164 patients (mean age 52 years, 69% female, median duration of IBS 17 years) were available at follow up, 143 patients (88%) had consulted their GP of whom 31 (19%) had consulted for IBS. Mean number of sick- leave days for IBS and comorbidity were 1.7 and 16.3 respectively (p < 0.01), costs related to IBS and comorbidity were 954 NOK and 14854 NOK respectively (p < 0.001). Age, organic diseases and somatic symptoms, but not IBS severity, were significant predictors for total costs.

**Conclusion:**

Costs for health resource use among patients with IBS in general practice were largely explained by comorbidity, which generated ten times the costs for IBS.

## Background

Irritable bowel syndrome (IBS) is a functional gastrointestinal disorder (FGID) affecting 8%-20% of the adult population in the western countries [1-3]. IBS is considered a significant burden for patients and society due to reduced quality of life and increased use of health resources [2,4,5]. Patients with IBS consume over 50% more health-care resources than matched controls without IBS [6]. A systematic review of studies addressing the direct and indirect costs of IBS in the US and UK included 18 of 24 identified publications between 1991 and 2003 [7]. Total direct cost estimates per person per year ranged from US Dollar (USD) 348 to USD 8750 and indirect costs ranged from USD 355 to USD 3344. Costs were influenced by a range of factors including age, consultation behaviour, comorbidity and severity of symptoms. The review recommended further studies to take these influential factors into account. Nevertheless, a common inference and argument for development of drugs towards symptoms of IBS is that the increased health care use and reduced quality of life is due to symptoms of IBS [8].

Consideration of comorbidity as an explanatory factor for use of health resources is warranted since patients with IBS frequently report other somatic and psychiatric disorders [9,10]. Such comorbidity might have a significant impact on health resource use and costs for patients with IBS. Most research on IBS has been performed on referred patients with IBS with questionable applicability in a general practice setting. Since most patients with IBS are managed in general practice, we aimed to measure health resource use and its costs in patients with IBS in general practice, and to explore the factors generating these costs.

## Methods

### Study design and material

This prospective cohort study was designed to identify and follow up a representative sample of patients with IBS (according to Rome II criteria) in Norwegian general practice. Of 3369 consecutive patients consulting their GP, 3092 (92%) answered a short questionnaire regarding abdominal complaints, prior to the consultation. Of 830 patients with abdominal complaints, for which they had consulted or wanted to consult their GP, 733 (89%) were assessed with the Rome II criteria for functional bowel disorders [11]. Of the 733 patients 278 patients had IBS according to the Rome II criteria. These 278 patients were invited to participate if the GP, based on all available information about the patient, agreed on the IBS diagnosis. A total number of 208 patients with IBS were included. Details about study design are given elsewhere [10]. The patients were invited by informed consent to further characterisation, by completing questionnaires at the first visit. After 6-9 months, included patients were invited to a follow up interview with repeated assessment of symptoms and detailed assessment of health resource use. No specific attention towards the abdominal complaints was to be given by the GP in the follow up period.

The study was performed during 2001in the county of Oppland in Norway, with 26 participating GPs working in nine health centres. In Norway, patients must seek health care through their locally assigned GP. Members of the practice staff (study nurses) performed the practical work related to the study. They were released from other duties, and trained to ensure satisfactory protocol adherence.

All patients were characterised at baseline for sociodemographic data and somatic and psychiatric comorbidity by self-completed questionnaires. Sociodemographic data included age, gender, smoking status, alcohol use, education (> 10 years), working status (employed, disability benefit, retired, students). IBS symptoms were assessed for severity and duration. Severity was measured as mild (with no interference of daily activities), moderate (with some interference, but no disruption of daily activities) and severe (with disruption of daily activities) with scores 1-3, and frequency as average number of days per week with abdominal pain/discomfort (0-1/2-3/4-5/> 5) with scores 1-4. An IBS symptom score was calculated by multiplying severity and frequency (range 1-12). Somatic diseases were measured by 12 questions about different organic diseases present within the 3 last months (yes/no) and presented as a sum-score (range 0-12). Somatic symptoms were measured by 17 questions about somatic symptoms from the Subjective Health Complaints (SHC) inventory [12]. The SHC inventory consists of a list of 29 common health complaints experienced the last 30 days, with the intensity of each complaint graded on a four point scale (not at all/little/some/severe). An SHC total score is created by adding the scores of each item. 12 questions about gastroenterological and psychiatric symptoms were excluded to avoid duplicate assessment, giving a SHC-17 score from 0 to 51. Psychiatric comorbidity was measured by Symptom Check List 10 (SCL-10, score range 1 - 4.0), which measures psychological distress, with a cut off point of > 1.85 as a valid predictor of mood disorder. Health anxiety was measured by Whitley index (score range 14-60) and neuroticism by EPQ-10 (score range 0-10) [13-15].

### Measurement of health resource use and associated costs

Health resource use and associated costs were measured for investigations, consultations, hospitalisations, medications, and alternative health care products and number of sick leave days was noted. Assessment was primarily done by structured questions in a Case Report Form (CRF) completed by study nurses at the follow up interview. All questions in the CRF separated use of health resources related to symptoms of IBS (IBS-related) from use of health resources related to other symptoms and diseases (comorbidity-related). Nurses were instructed to examine the electronic medical records (EMR) to identify relevant information. If information was not available in the EMR, data were assessed by patients recall. Since follow up time varied between six and nine months, only the last six months of the follow up period was used to measure use of health resources. The cost analysis was restricted to direct medical costs for health resource use and did not include sick-leave days, which we considered an indirect cost.

Investigations were assessed only for IBS and included blood samples, stool tests, endoscopic procedures and medical imaging by x-ray, CT, MRI and ultrasound. Costs for consultations and investigations were calculated using National Tariffs for the Public Health Service for year 2001, in Norwegian kroner (NOK, €1 equals NOK 8). Consultations were measured as the number of visits to GPs, specialists, chiropractors, physiotherapists, psychotherapists and alternative health care providers. Hospitalisations were measured as numbers of hospital stays for each patient. Costs for hospitalisations were calculated using Diagnosis Related Groups (DRG) and national tariffs. Medications were measured as total use of drugs by physicians' prescription and drugs purchased over the counter. Medication costs were calculated by The Norwegian Pharmaceutical Product Compendium for year 2001. Costs for on demand medications were calculated using the lowest dose 3 times per week. Alternative health care products and their costs were measured and estimated by patients recall. Sick-leave days were measured directly by retrieving data from the EMR.

### Data analysis and statistics

Data were analysed by descriptive statistical methods in SPSS v. 15. Use of health resources and its costs were analysed by univariate, bivariate and multivariate analyses. Non-parametric tests (eg. Wilcoxon, Spearman's rho and Mann Whitney U test) were performed in the cost analyses due to skewed distribution of costs as dependent variables. Variables with p < 0.2 in the bivariate analyses were entered in multivariate lineary regression analyses. Age and gender were kept as sociodemographic variables in the multivariate analysis, regardless of statistical significance in bivariate analysis, and the dependent variables (costs) were log 10 transformed.

### Ethics

The study was performed according to the Declaration of Helsinki and approved by the Regional Committee for Medical Research Ethics and the Data Inspectorate, Oslo, Norway.

## Results

### Characteristics of patients

Table 1 gives the characteristics of the 164 patients who were available at the follow up after 6-9 months and included in the analyses. The 44 patients lost to follow-up (36-unwilling or no contact, 6- organic diseases diagnosed and 2- missing data in CRF) did not differ significantly from patients included in the analyses for any of the baseline characteristics (data not shown).

**Table 1:** Characteristics of 164 patients with IBS included in the analyses.

Variables	
Age (years), mean (SD)	52 (15.1)
Female gender	69%
Gastro-esophageal reflux (Rome II definition)	43%
Dyspepsia (Rome II definition)	9%
Duration IBS complaints (> 1 year)	85%
Duration IBS in years, mean (SD)	17 (15)
IBS-severity (mild/moderate/severe)	36%/52%/12%
Education > 10 years	63%
Mood disorder (SCL 10 > 1.85)	37%
Number of organic diseases, mean (SD)	2.0 (1.8)
Smokers	35%
Alcohol use (> 2 occasions/week)	18%
SHC-17, mean (SD)	13.2 (8.2)
EPQ, mean (SD)	4.0 (2.9)
Whitley, mean (SD)	25.9 (8.2
SCL-10, mean (SD)	1.8 (0.6)

Results are reported as percentages if not otherwise specified.

### Use of health resources and associated costs

In the 6 month follow up period, 143 patients (88%) consulted their GP of whom 31 (19%) consulted for IBS-complaints. The mean number of visits was 3.6 (SD 2.9). As for investigations for IBS-complaints in the study period, blood tests were performed in 24 patients (15%), endoscopy in 8 patients (5%) and barium enema in14 patients (9%). Medications were used by 146 patients (89%) of whom 14 (9%) used medications for symptoms of IBS. Sick-leave days related to IBS were significantly lower than related to comorbidity (mean 1.7 days (SD 16) versus 16.3 days (SD 43), p < 0.001).

Table 2 gives the costs of health care in the 6 month follow up period. Median total costs related to symptoms of IBS and comorbidity were NOK O (0-59728) and NOK 3190 (range 0-193355) respectively. The median costs for investigations related to IBS were NOK O (range 0-2170).

**Table 2:** IBS and comorbidity-related costs of health care measured in Norwegian Kroner (NOK) in the six-month follow-up period.

Type of health resource	IBS-related costs	Comorbidity-related costs	p-values *
Consultations	169 (610) *0 (0-5520)*	1315 (1915) *539 (0-12688)*	P < 0.001
Hospitalisations	710 (6413) *0 (0-58260)*	10302 (27330) *0 (0-174780)*	P < 0.001
Medications	47 (261) *0 (0-2785*)	2999 (6796) *1544 (0-74634*)	P < 0.001
Alternative products	26(127) *0 (0-1110)*	238(544) *5 (0-4000*)	P < 0.001
Total	1049(6574) *0 (0-60468*)	14856(30570) *3190 (0-193355*)	P < 0.001

* Wilcoxon test due to skewed data.

Results are given both as mean (SD) and median with range (*italic*). €1 equals NOK 8.

### Factors associated with and predicting costs for health resource use

Table 3 and 4 give the associations (shown as bivariate and multivariate analyses) between the background variables and costs. Age, organic diseases and somatic symptoms were significant independent predictors for total costs; mood disorder was a significant predictor for the use of alternative products. IBS symptom score (severity and frequency) was not associated with any of the cost variables included IBS-related costs.

**Table 3:** Associations between background variables and costs of health care in the six-month follow up period.

Variables	Costs for consultations	Costs for hospitalisations	Costs for medications	Costs for alternative products	Total costs	Total IBS related costs
Age	0.841	0.579	**0.001**	0.182	**0.003**	0.498
Gender (female)	0.692	0.612	0.610	0.125	0.965	0.192
Education > 10 yrs	0.406	0.465	**0.008**	0.126	0.251	0.929
Smoker	0.631	0.725	0.607	0.301	0.567	0.531
Alcohol use	0.687	**0.046**	0.854	0.834	0.552	0.813
Reflux/Dyspepsia	0.261	0.543	0.309	0.388	0.385	0.427
IBS-symptom score	0.148	0.767	**0.001**	0.838	0.075	0.059
Somatic symptom score (SHC-17)	**0.001**	0.713	**0.001**	**0.002**	**0.001**	0.524
Organic disease score	**0.003**	**0.014**	**0.001**	0.434	**0.001**	0.914
Neurotissism	0.151	0.796	0.076	**0.006**	0.224	0.288
Health anxiety	**0.008**	0.873	**0.024**	0.105	0.132	0.892
Mood disorder (SCL 10)	0.292	0.975	**0.050**	**0.001**	0.140	0.338

The results are given as p-values with significant values in bold. For all background variables higher values were associated with increased costs.

**Table 4:** Multivariate analyses of variables associated with costs of health care (p < 0.20) in bivariate analysis.

Costs	Age	Gender	Organic diseases	Somatic symptoms	Mood disorder	IBS symptom score	Alcohol use
Costs for consultations	ns	ns	β = 0.133 p = 0.002	ns	ns	ns	ns
Costs for hospitalisations	ns	ns	β = 0.184 p = 0.031	ns	ns	ns	β = 0.915 p = 0.025
Costs for medications	β = 0.032 p = 0.001	ns	ns	β = 0.031 p = 0.002	ns	ns	ns
Costs for alternative products	β = 0.013 p = 0.052	ns	ns	ns	β = 0.484 p = 0.004	ns	ns
Total costs	β = 0.013 p = 0.008	ns	β = 0.090 p = 0.047	β = 0.021 p = 0.034	ns	ns	ns
Total costs for IBS	ns	ns	ns	ns	ns	ns	ns

Results are given as β and p-values if statistically significant associations were found (p < 0.05) or as non-significant (ns).

## Discussion

### Main findings

This study found that costs for health care generated by patients with IBS were mainly driven by comorbidity. Our finding that comorbid disorders generated approximately ten times the costs for IBS was consistent for consultations, hospitalisations, medications and use of alternative health care products. A similar pattern was found for sick leave days although we did not compute costs for this dimension of health resource use. We are not aware of other studies demonstrating a similar difference in IBS-related and comorbidity-related use of health resources. Another noteworthy finding was the limited use of health resources related to symptoms of IBS. Less than one third of patients reported consultations, investigations or the use of medications for IBS during the six-month follow-up period. In contrast, patients on average consulted their GP nearly four times and reported 17 sick leave days in the same period of time. As we have published earlier, our sample of patients were characterized by high levels of somatic and psychiatric comorbidity [10]. Importantly, the total direct costs for health care generated by our sample lie within the range of costs reported in the systematic review of costs for IBS in the US and UK [7]. Taken together the findings suggest that from a health economic perspective symptoms of IBS constitute a minor problem but patients with IBS constitute a major problem. Patients with IBS referred to specialists exhibit more severe IBS symptoms than patients handled in general practice [16]. Since these patients also exhibit higher levels of comorbidity than patients in general practice it would be useful to study the impact of comorbidity on health resource use in secondary health care [9]. This was partly investigated in a study of referred patients with severe IBS in the UK [17]. Interestingly, abdominal and psychological symptoms were independently associated with impaired health-related quality but the authors could not find predictors for the observed excess health care costs and loss of productivity. Given the findings in our study one likely predictor would be the somatic comorbidity, which was not included as an explanatory factor in the UK study.

Knowledge about predictors of health care costs in patients with IBS can help target interventions to reduce such costs. Our multivariate analyses revealed age, somatic comorbidity (ie. number of organic diseases and somatic symptoms) and psychiatric comorbidity as main predictors of different domains of health care costs. The individual predictive values of these factors are limited by the significant correlation between somatic and psychological comorbidity in this sample of patients (r^2 ^= 46%) [10]. Accordingly, bivariate analyses revealed several somatic and psychiatric comorbidity-variables to be associated with most domains of health care costs. In contrast to what has been reported by Longstreth et al, severity of IBS symptoms did not predict costs for IBS-related health care or total costs for health care in our study [18]. This discrepancy is most likely explained by our use of multivariate analyses to control for the confounding effects of comorbidity on health care costs. Other possible explanations include our crude measurement of IBS severity, the limited sample size and our sampling of patients with long standing symptoms who did not necessarily primarily consult for symptoms of IBS at the time they were included in this study. The latter possible explanation is reflected in the observed low number of investigations performed for IBS and the low median costs for medications for IBS. If we had included only patients with recently onset severe symptoms of IBS the costs for IBS-related health care would likely have been more impressive. The finding that increasing age independently predicted health care costs could be explained by sociodemographic factors or comorbidities (ie. more severe diseases and/or longer stays in hospitals) not captured by our analyses.

The considerable impact of comorbidites on health resource use observed in our study is unlikely to be unique to patients with IBS. A fairly similar pattern would probably emerge if we had included patients with a primary diagnosis of for example chronic low back pain, fibromyalgia or chronic fatigue syndrome instead of IBS as the qualifying diagnosis. Accordingly a systematic review of patients with chronic low back pain found an association between comorbid mood disorders and health care costs [19]. More recently a study of 16 567 patients with chronic low back pain reported that physical and mental health comorbidities were associated with healthcare utilization and costs [20]. The findings highlight the need to maintain a wide focus not restricted to specific diagnoses both in clinical research and practice for patients with a variety of somatic and psychiatric illness.

### Strengths and limitations

Among the strengths of this study are the cohort study design, the high follow up rate and the meticulous retrieval of data from the EMR concerning consultations, hospitalisations, medications and sick-leave days. Another strength is that the IBS diagnosis was based on the internationally recognised Rome II-criteria designed for research purposes [11]. The Rome II criteria have now been superseded by the Rome III criteria but only minor changes have been made regarding the diagnosis of IBS. A weakness of our study and possible source of information error is that the use of medications over the counter, alternative health care and presence of organic diseases were assessed by patients recall. Another possible source of information error is that study nurses who were aware of the objectives in the study and responsible for collecting data at follow up could have influenced categorization of health resources as related to IBS or related to comorbidity. Another potential weakness is that we can not determine to what extent patients with IBS in our study used more health resources than patients in general practice without IBS. However, this has been firmly established by others [7].

## Conclusion

It seems reasonable to conclude that effective treatment targeting IBS symptoms alone will have limited impact on the use of health resources generated by these patients in a general practice setting. Our findings call for research to establish the impact of comorbidity on use of health resources and quality of life in different clinical settings. We suggest that future research to improve treatment for patients with IBS should take into consideration comorbidity as a main driver for health resource use and reduced quality of life [21]. The comorbidity of IBS has implications for choice of interventions (e.g mind body intervention) and outcomes in design of treatment trials [22,23]. Regarding choice of outcomes we suggest that both somatic and psychiatric comorbidities are included as patient-important outcomes together with symptoms of IBS, quality of life and use of health resources in future trials. For clinicians who encounter patients with IBS in clinical practice our study pinpoints the importance of a holistic approach recommended in recent clinical guidelines [24,25].

## Abbreviations

IBS: Irritable Bowel Syndrome; FBD: Functional Bowel Disorder; EMR: Electronic Medical Records; GP: General Practitioner; NOK: Norwegian Kroner.

## Competing interests

The study was funded in part through a grant from Novartis Pharmaceuticals in 2001.

## Authors' contributions

All authors except PAJ contributed to study design and data collection. All authors were involved in drafting the analysis plan and interpretation of the data. PAJ analyzed the data and was principal author of the manuscript. All authors contributed to manuscript drafting and revision and approved the final manuscript.

## Pre-publication history

The pre-publication history for this paper can be accessed here:

http://www.biomedcentral.com/1471-230X/10/31/prepub
